# Tissue analyses reveal a potential immune-adjuvant function of FAP-1 positive fibroblasts in non-small cell lung cancer

**DOI:** 10.1371/journal.pone.0192157

**Published:** 2018-02-07

**Authors:** Thomas Karsten Kilvaer, Mehrdad Rakaee, Turid Hellevik, Arne Østman, Carina Strell, Roy M. Bremnes, Lill-Tove Busund, Tom Dønnem, Inigo Martinez-Zubiaurre

**Affiliations:** 1 Department of Oncology, University Hospital of Northern Norway, Tromsø, Norway; 2 Department of Clinical Medicine, UiT The Artic University of Norway, Tromsø, Norway; 3 Department of Medical Biology, UiT The Arctic University of Norway, Tromsø, Norway; 4 Department of Oncology-Pathology Cancer Center Karolinska, Karolinska Institutet, Stockholm, Sweden; 5 Department of Clinical Pathology, University Hospital of Northern Norway, Tromsø, Norway; University of Bergen, NORWAY

## Abstract

**Objectives:**

Selective targeting of cancer-associated fibroblasts (CAFs) has been proposed to synergize with immune-checkpoint inhibitors. While the roles of CAFs in cancer development are well described, their immune-regulatory properties remain incompletely understood. This study investigates correlations between CAF and immune-markers in tumor stroma from non-small cell lung cancer (NSCLC) patients, and examines whether a combination of CAF and immune cell scores impact patient prognosis.

**Methods:**

Tumor specimens from 536 primary operable stage I-III NSCLC patients were organized in tissue microarrays. Expression of protein-markers was evaluated by immunohistochemistry.

**Results:**

Fibroblast and stromal markers PDGFRα, PDGFRβ, FAP-1 and vimentin showed weak correlations while αSMA, and Masson’s trichrome did not correlate with any of the investigated markers. Hierarchical clustering indicated the existence of different CAF-subsets. No relevant correlations were found between any CAF-marker and the immune-markers CD3, CD4, CD8, CD20, CD68, CD1a, CD56, FoxP3 and CD45RO. High density of fibroblast-activation protein positive mesenchymal cells (CAF^FAP^) was associated with better prognosis in tumors with high infiltration of CD8 and CD3 T-lymphocytes.

**Conclusions:**

The presented data suggest that CAFs, irrespective of identity, have low influence on the degree of tumor infiltration by inflammatory- and/or immune-cells. However, CAF^FAP^ may exert immuno-adjuvant roles in NSCLC, and targeting CAFs should be cautiously considered.

## Introduction

In solid tumors, complex and reciprocal interactions between neoplastic cells and surrounding cells lead to a tumor tissue compartment often referred to as reactive stroma, desmoplastic stroma or tumor microenvironment. A dominant component of the tumor stroma are fibroblasts, which are known to play determinant roles in tumor initiation, expansion, dissemination and metastasis [1]. Cancer-associated fibroblasts (CAFs) is a generic name given to a heterogeneous group of non-epithelial, non-immune cells with a likely mesenchymal lineage, located within tumors or at the tumor borders [2]. Quiescent connective tissue fibroblasts are generally considered indolent, exhibiting rather low metabolic and transcriptomic activity, and expressing classical mesenchymal markers such as vimentin, integrin α1β1 or FSP-1 [3]. Fibroblasts associated with tumors normally display an activated phenotype, and depending on their origin, morphology or spatial distribution, they may receive different names such as myofibroblasts, activated tumor fibroblasts, activated stellate cells, bone marrow-derived mesenchymal stromal cells or pericytes [4, 5]. Several markers such as αSMA, FAP-1, desmin, podoplanin, neuron-glial antigen 2 (NG2) and PDGF receptors-α and -β are used to identify CAFs. However, due to the great plasticity of this cell population, none of these markers can be used as a universal marker for all CAFs as their expression is likely to be temporal and context dependent [6]. Different CAF subsets, expressing overlapping and non-overlapping markers, can be identified in a single tumor. However, it remains unknown whether the various CAF subtypes in tumors have different functions. In non-small cell lung cancer (NSCLC), several studies have explored the prognostic significance of established CAF markers such as podoplanin, vimentin, FAP-1, αSMA or PDGFRβ. In most cases, these markers have demonstrated unfavorable outcomes related to survival (Table 1).

**Table 1:** Prognostic role of different CAF markers in lung cancer cohorts.

Fibroblast marker	Tumor subtype	Samples (n)	Technique	Prognostic impact	Ref.
**αSMA**	NSCLC Stage I-III	78	IHC whole tissue/PCR	Unfavorable	Chen [7]
	NSCLC Stage I-IIIA	536	IHC/TMA	No significance	Kilvaer [8]
**αSMA/ Vimentin**	ADC	102	IHC whole tissue	Unfavorable	Shu [9]
**Vimentin**	NSCLC Stage I-IIIA	335	IHC/TMA	No significance	Al-Saad [10]
**Podoplanin**	ADC Stage-I	304	IHC whole tissue	Unfavorable	Ito [11]
	ADC	177	IHC whole tissue	Unfavorable	Kawase [12]
	ADC (post chemotherapy)	87	IHC whole tissue	Unfavorable	Koriyama [13]
	ADC N2 Stage-III	112	IHC Lymph nodes	Unfavorable	Neri [14]
	SCLC	36	IHC whole tissue	Favorable	Takahashi [15]
	NSCLC	400	IHC/TMA	Unfavorable	Kitano [16]
	SCC Stage-I	142	IHC whole tissue	Unfavorable	Ono [17]
**FAP-1**	NSCLC	59	IHC whole tissue	Unfavorable	Liao [18]
	NSCLC Stage I-III	536	IHC/TMA	Favorable in SCC	Kilvaer [8]
**MMP-2**	NSCLC Stage I-IIIA	218	IHC whole tissue	Unfavorable	Ishikawa [19]
	NSCLC	212	IHC whole tissue	Unfavorable	Leinonen [20]
**CD99**	NSCLC	190/240 (2cohorts)	IHC/TMA	Favorable	Edlund [21]
**PDGFRβ**	NSCLC	190	IHC/TMA	No significance	Edlund [21]

Abbreviations: NSCLC, non-small cell lung cancer; IHC, immunohistochemistry; PCR, polymerase chain reaction; ADC, adenocarcinoma; SCLC, small-cell lung cancer; SCC, squamous cell carcioma

Despite the widely acknowledged role of CAFs in malignant progression, the understanding of their relationship with tumor-infiltrating lymphocytes (TILs) is incomplete. Emerging evidence propose that the tumor stroma influence tumor immunity and response to immunotherapy [22]. CAFs are suggested to interfere with tumor immunity in multiple ways, including remodeling of extracellular matrix, shaping the phenotype of vessels and endothelial cells and directly influencing the migration and function of inflammatory and immune cells by the release of paracrine signals. In general, CAFs are believed to exert pro-inflammatory and immunosuppressive functions in tumors [23, 24]. However, some studies have challenged this view, arguing that different CAF subsets may exert opposite functions, and that in a context-dependent manner, CAFs may aid the antitumor immune responses [25]. In the context of cancer immunotherapy, selective depletion of CAF^FAP^ has been shown to synergize with immune-checkpoint inhibitors in pre-clinical models [26], thus proposing that targeting immunosuppressive elements of the tumor stroma may aid to the efficacy of immuno-therapeutic interventions.

We have previously explored the prognostic significance of CAF-markers in the stroma of resected NSCLC tumors [8], with the intriguing finding that high CAF^FAP^ levels were associated with an improved prognosis in patients with squamous cell carcinoma. Earlier studies on NSCLC have also highlighted the prognostic relevance of other stromal markers such as CD99 [21], Forkhead Box F1 [27], and Cox-2 [28]. In the present study, we explore associations between CAFs and immune cell infiltrates in NSCLC tumor tissues. Additionally, we investigate whether CAFs impact patient survival according to different levels of immune cell infiltration.

## Materials and methods

### Patients and clinical samples

This study presents data on a cohort of 536 unselected stage IA-IIIA NSCLC patients. A detailed description of the cohort was published previously by our group [29]. Briefly, the included patients were staged after the 7^th^ edition of the UICC TNM classification [30], and histologically classified according to the 2013 edition of the pathological classification of lung cancer [31]. Of the 536 patients, 289 were squamous cell carcinomas, 201 were adenocarcinomas and 46 were classified as NOS. Most patients classified as NOS were previously considered as large cell carcinoma, while a few patients were too undifferentiated to be classified. The Regional Committee for Medical and Health Research Ethics (REK-Nord) approved the use of human material for this study (Project-ID: 2016/2307/REK-Nord). Due to the retrospective nature of the study, and the fact that two thirds of the study population was deceased at time of study initiation, the need of written informed consent was waivered. All methods involving human material were performed in accordance with relevant guidelines and regulations.

### Tissue micro-array construction, immunohistochemistry and scoring

**Fig 1:**
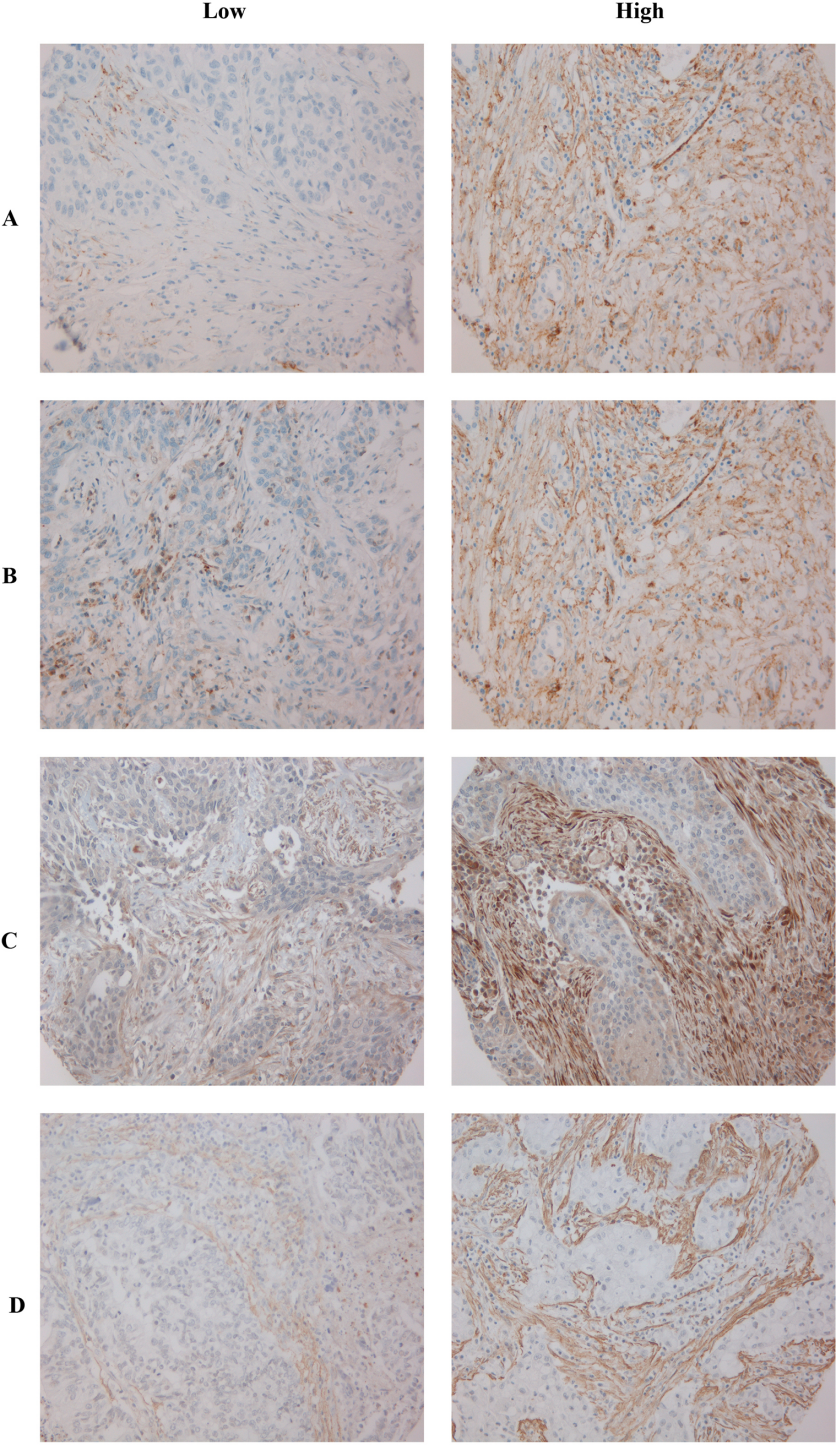
Immunostaining of TMA cores showing low vs high score of the four main CAFs markers. (**A)** PDGFRβ; (**B)** PDGFRα; (**C)** FAP-1; (**D)** αSMA. Abbreviations: PDGFR, platelet-derived growth factor receptor; FAP-1, Fibroblast activating protein 1; αSMA, alpha-smooth muscle actin.

The work-flow for sample preparation, tissue micro-array (TMA) construction, immunohistochemistry (IHC) and scoring of TMA-slides is extensively documented [8, 29, 32]. In brief, representative areas of primary lung tumor stroma specimens were identified on H&E slides. Two duplicate tissue cores were collected from the primary tumor blocks based on the overlay of the H&E stained slides. TMAs were assembled using a Beecher Instruments tissue-arraying instrument (Beecher Instruments, Silver Springs, MD, USA). Blocks were sectioned with 4 μm thickness and heated over-night at 60°C. The IHC staining procedures- including validations of antibodies—for FAP-1 and αSMA [8], CD3 (pan T-cell marker), CD4 (T-helper cells), CD8 (cytotoxic T-cells), CD20 (B-cells) and CD45RO (T-memory cells) and CD1A (Dendritic cells), CD56 (Natural Killer cells) and CD68 (macrophages) [33] were previously reported. The S1 Table includes a summary of staining procedures for the CAF markers [29]. Expert pathologists established semi-quantitative cut-offs for each marker. Immune- and CAF markers were scored as percentages of positive cells from the total number of cells in the stromal area, using the following thresholds: CD3, CD4, CD8, CD20 and CD45RO 0, = <1%, 1 = 1–5%, 2 = 6–25%, 3 = 26–50%, 4 = >50% (29); CD1A and CD56, 0 = < 1%, 1 = > 1%; CD68, 0 = <25%, 1 = > 25% [33]; FAP1, [SMA, PDGFRα and PDGFRβ 0 = <1%, 1 = 1–10%, 2 = 11–50%,d 3 = > 50% [8]. Vimentin was scored based on intensity and density with the following thresholds: intensity 1 = weak, 2 = intermediate and 3 = strong [10]. Micro vessels density and lymphatic micro vessel density was scored based on the total number of vessels found per core, using the following thresholds: CD34, 0 = negative; 1 = (1–10 vessels per core); 2 = (11–20 vessels per core); 3 = (>20 vessels per core) [30]; D240, 0 = negative; 1 = (one vessel per core); 2 = (2–5 vessels per core); 3 = (>5 vessels per core) [34]. All TMA cores were scored independently by two investigators, blinded to each other and to patient data. High expression of CAF-markers were defined as: > 0.5 (FAP-1), >2 (αSMA), >1,75 (PDGFRα) and > 1.5 (PDGFRβ) utilizing an optimal cut-off approach (Fig 1). The cut-offs used for FAP1 and αSMA were previously defined [8]. In order to evenly distribute CAF high and low patients in subgroup analyses based on immune cell infiltration, high expression of immune markers (CD3 and CD8) was decided bases on a median cut-off approach (S1 Fig)

### Statistical methods

All statistical analyses were conducted in RStudio, version 1.0.136 with R version 3.3.2 and packages "sjmisc", "Hmisc", "survival", "ggplot2", "reshape2", "grid", "gridExtra" and "cowplot". Associations between the dichotomized markers and clinicopathological variables were tested with Chi-square or Fisher's exact tests. Due to the semi-quantitative scoring of the investigated markers, Spearman`s rank-correlation was used to examine the between-marker correlations and visualized utilizing heat-maps and hierarchical clustering. Survival analyses were conducted and visualized using the log-rank test and the Kaplan-Meier method To allow the investigation of markers in the presence of other co-variables from the same dataset, Cox proportional hazards models were fitted to the data. A supervised iterative process, based on significance levels in each step, was used to select the variables included in the final models. The significance level used for all statistical tests was P < 0.05.

## Results

### Associations between markers and clinicopathological variables

No significant associations were observed between neither PDGFRα, nor vimentin expression and clinicopathological variables (Table 2). Low expression of PDGFRβ was associated with normal ECOG status (P = 0.003) and high expression of αSMA was associated with vascular invasion (P = 0.012). No associations between FAP-1 and clinicopathological variables was observed, as previously reported by our group [8].

**Table 2:** Correlations between marker expressions and clinicopathological variables with stromal PDGFRα, PDGFRβ, αSMA and vimentin (chi-square and Fisher's exact tests).

	PDGFRα S			PDGFRβ S			αSMA			Vimentin		
	Low	High	*P*	Low	High	*P*	Low	High	*P*	Low	High	*P*
**Age**			0.431			0.443			0.341			0.594
<65	146	68		124	90		148	79		100	35	
≥65	207	81		176	109		214	94		133	55	
**Gender**			0.225			1.000			0.138			0.331
Female	109	55		97	64		123	47		51	25	
Male	244	94		203	135		239	126		182	65	
**Weight-loss**			0.659			0.119			0.602			0.808
<10%	319	132		275	173		327	153		211	80	
>10%	34	17		25	26		35	20		22	10	
**Smoking status**			0.842			0.100			0.726			0.041
Never	10	4		12	2		10	7		13	0	
Present	222	98		184	132		233	109		146	63	
Previous	121	47		104	65		119	57		74	27	
**ECOG**			0.756			0.003			0.704			0.860
Normal	203	91		193	98		211	98		136	51	
Slightly	125	48		91	82		125	65		85	33	
In bed<50%	25	10		16	19		26	10		12	6	
**Histology**			0.069			0.093			0.393			0.303
SCC	190	83		152	120		198	90		137	53	
ADC	127	60		124	64		137	64		77	25	
NOS	36	6		24	15		27	19		19	12	
**T-stage**			0.159			0.840			0.505			0.888
T1a	49	22		45	26		53	21		25	11	
T1b	68	20		54	34		63	31		42	15	
T2a	127	49		101	71		128	61		86	36	
T2b	42	29		46	25		48	27		33	14	
T3	64	26		51	40		68	29		47	14	
T4	3	3		3	3		2	4		0	0	
**N-stage**			0.273			0.911			0.541			0.227
N0	237	105		202	136		248	116		159	64	
N1	85	27		68	42		75	42		58	16	
N2	31	17		30	21		39	15		16	10	
**P-stage**			0.769			0.938			0.875			0.954
IA	93	34		79	47		94	40		53	21	
IB	79	33		67	43		81	41		52	23	
IIA	82	32		65	45		76	42		63	21	
IIB	48	23		43	29		51	24		35	14	
IIIA	51	27		46	35		60	26		30	11	
**Differentiation**			0.129			0.250			0.688			0.917
Poor	153	59		131	78		160	70		98	39	
Moderate	152	77		138	91		158	82		104	38	
Well	48	13		31	30		44	21		31	13	
**Vascular invasion**			0.520			0.906			0.012			0.236
No	292	119		244	163		306	130		200	72	
Yes	60	29		55	35		55	42		33	18	

Abbreviations: PDGFR, platelet derived growth factor; αSMA, alpha-smooth muscle actin; S, stroma; ECOG, Eastern cooperative oncology group; SCC, squamous cell carcinoma; ADC, adenocarcinoma; NOS, not otherwise specified

### Correlations between CAF markers

**Fig 2:**
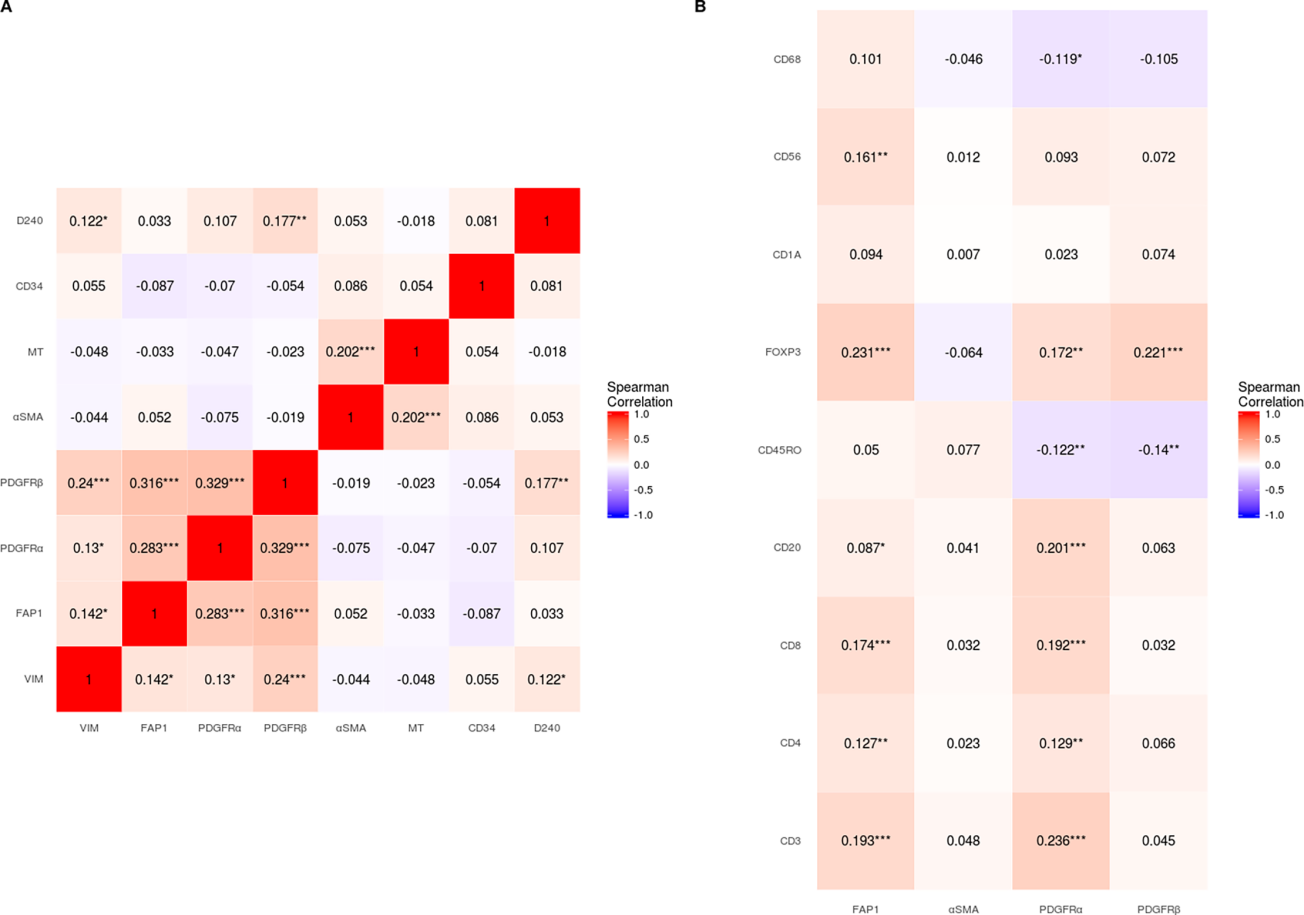
**Spearman's rank correlations** between **A)** Different CAF markers and **B)** CAF markers and markers of leukocyte subsets. *P < 0.05, **P < 0.01, ***P < 0.001. Abbreviations: CAF, cancer-associated fibroblast; Vim, vimentin; FAP-1, Fibroblast activating protein 1; PDGFR, platelet-derived growth factor receptor; αSMA, alpha-smooth muscle actin; MT, Masson's trichrome; CD, cluster of differentiation.

**Fig 3:**
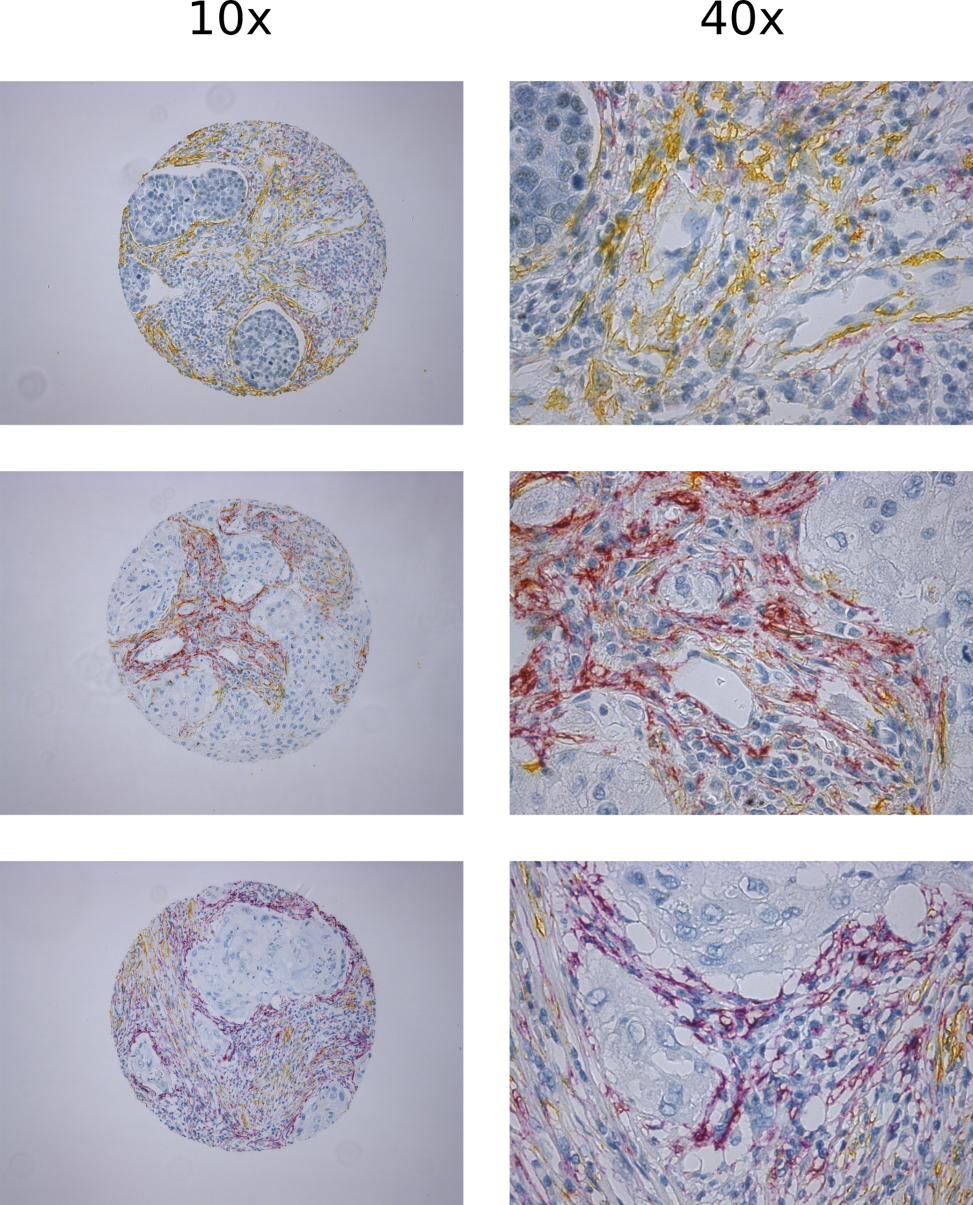
Double staining of FAP-1 & αSMA showing distinct expression patterns: in three randomly selected TMA cores corresponding to three NSCLC patients co-stained with FAP-1(purple) and αSMA(yellow).

Fig 2A and S2A and S2B Fig summarizes the correlations between different CAF-markers investigated in the overall cohort and the two main histological subgroups of NSCLC, respectively. In the overall cohort, hierarchical clustering suggested a CAF phenotype in patients expressing vimentin, FAP-1 and PDGFRα and -β. On the other hand αSMA was only weakly associated with the other CAF-markers (-0.2<R<0.2). Considering correlations with R-values < -0.2 and > 0.2: **1)** PDGFRα was associated with FAP-1 (R = 0.28) and PDGFRβ (R = 0.33); **2)** PDGFRβ was associated with vimentin (R = 0.24) and FAP-1 (R = 0.32); and **3)** αSMA was associated with Masson's trichrome (MT) (R = 0.20). In addition, correlations of CAF-markers with angio- and lymphangigenic markers CD34 and D240 had low R-values (< 0.2). Other significant, but weak (-0.2 < R < 0.2) associations were also observed. Correlations in histological subgroups were mostly similar to those observed in the overall cohort (S2A and S2B Fig). Distinct expression patterns of FAP-1 and αSMA (both considered hallmarks of activated CAFs) in the same tissue specimens are shown in Fig 3.

### Correlations between CAFs and immune-cell markers

Fig 2B and S2C and S2D Fig summarizes the correlations between CAF- and immune-cell markers in the overall cohort and the two main histological subtypes of NSCLC, respectively. No clear strong correlations between CAF- and immune-markers were observed. Considering correlations with R-values <-0.2 and > 0.2; **1)** FAP-1 was positively associated with FOXP3 (R = 0.231); **2)** PDGFRα was positively associated with CD3 (R = 0.236) and CD20 (R = 0.201) and **3)** PDGFRβ was positively associated with FOXP3 (R = 0.221). Other significant, but weak (R < 0.2) associations between CAF- and immune-markers was observed. Correlations in histological subgroups were mostly similar and similar to those of the overall cohort (S2C and S2D Fig). Images of TMA cores representing tumors with different scores of CD3+ and CD8+ cells are shown in S1 Fig.

### Survival analyses

**Fig 4:**
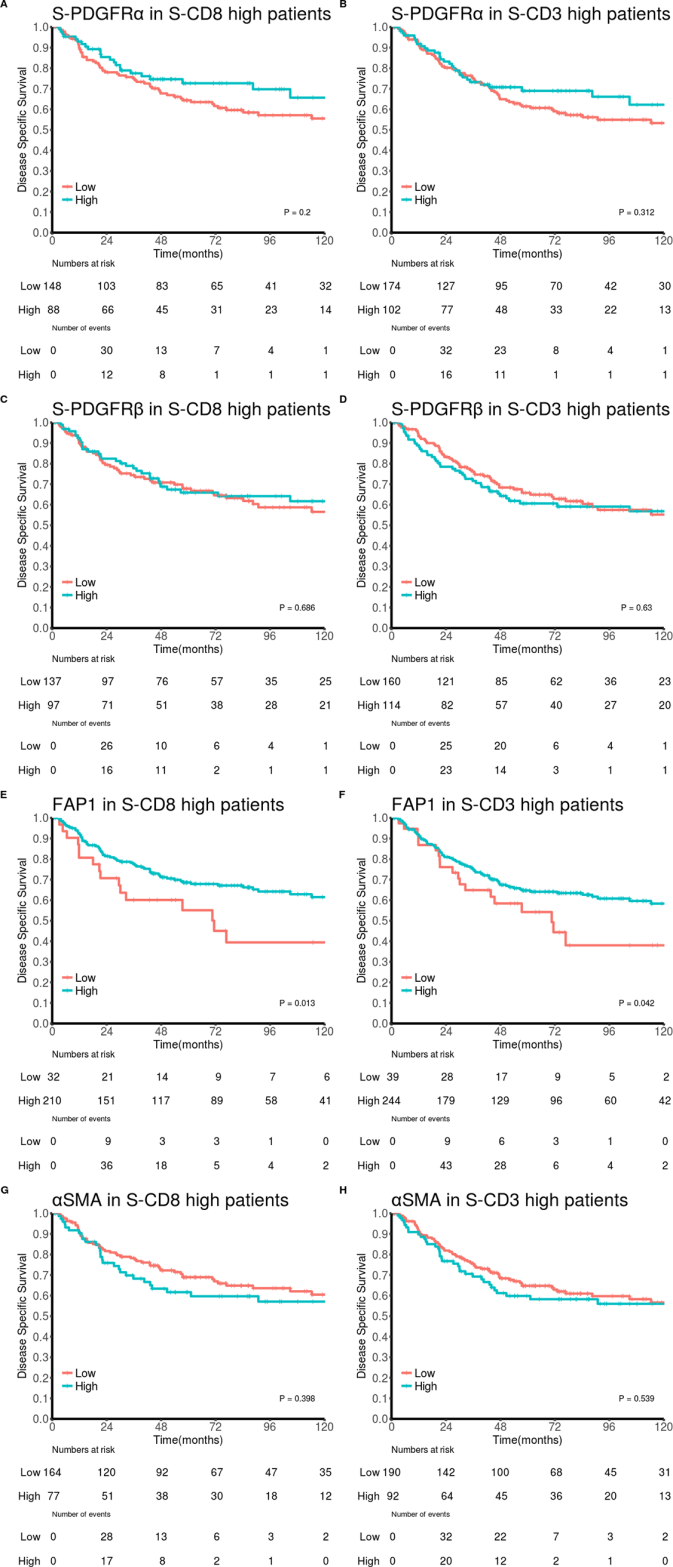
**Disease-specific survival curves** for: PDGFRα in patients expressing high levels of **A)** CD8 and **B)** CD3, PDGFRβ in patients expressing high levels of **C)** CD8 and **D)** CD3, FAP-1 in patients expressing high levels of **E)** CD8 and **F)** CD3 and αSMA in patients expressing high levels of **G)** CD8 and **H)** CD3. Abbreviations: FAP-1, Fibroblast activating protein 1; PDGFR, platelet-derived growth factor receptor; αSMA, alpha-smooth muscle actin.

Fig 4 and S3 Fig shows the survival curves of PDGFRα, PDGFRβ, FAP-1 and αSMA in patients expressing high and low levels of CD8 and CD3 respectively. In univariate analyses, high expression of FAP-1 was a significant positive marker for survival in patients with high expression of CD8 (*P* = 0.013) and CD3 (P = 0.042), while high expression of PDGFRβ was a significant negative marker for survival in patients with low expression of CD8 (*P* = 0.005) and near significant in patients with low expression of CD3 (*P* = 0.052).

In multivariable analyses, summarized in Table 3, FAP-1 was an independent positive marker for survival in patients with high, but not low, expression of CD8 (HR 0.42, 95% CI 0.24–0.74, *P* = 0.003, Table 3A) and PDGFRβ was an independent negative marker of survival in patients with low, but not high, expression of CD8 (HR 1.85, 95% CI 1.28–2.66, *P* < 0.001, Table 3E). Neither FAP-1, nor PDGFRβ were independent markers for survival in patients with high or low expression of CD3 (Table 3C, 3D, 3G and 3H).

**Table 3:** Multivariable models of FAP1 (A-D) and PDGFRβ (E-H) predicting survival of NSCLC patients with high and low expression of CD8 (A, C, E and G) and CD3 (B D, F and H, Cox regression analysis).

	**(A) CD8 High**		**(B) CD3 High**		**(C) CD8 Low**		**(D) CD3 Low**	
	**HR** **(95% CI)**	***P***	**HR** **(95% CI)**	***P***	**HR** **(95% CI)**	***P***	**HR** **(95% CI)**	***P***
**FAP1**								
Low	1		1		1		1	
High	0.42(0.24–0.74)	0.003	0.75(0.45–1.25)	0.266	0.99(0.65–1.49)	0.954	0.84(0.54–1.31)	0.452
**Gender**								
Female	1		1		1		1	
Male	2.3(1.37–3.85)	0.002	1.84(1.18–2.84)	0.007	1.48(0.98–2.23)	0.063	1.3(0.79–2.12)	0.302
**Histology**								
SCC	1		1		1		1	
ADC	3.13(1.91–5.16)	<0.001	2.25(1.45–3.48)	<0.001	1.16(0.78–1.73)	0.451	1.11(0.72–1.71)	0.647
NOS	1.92(0.76–4.88)	0.170	2.24(1.12–4.48)	0.022	0.96(0.51–1.8)	0.894	0.56(0.24–1.27)	0.166
**ECOG**								
Normal	1		1		1		1	
Slightly reduced	1.98(1.21–3.23)	0.006	1.36(0.89–2.08)	0.157	1.29(0.9–1.87)	0.171	1.53(1.02–2.29)	0.040
In bed <50%	2.92(1.11–7.65)	0.029	1.86(0.83–4.2)	0.133	1.36(0.62–2.99)	0.447	1.08(0.41–2.82)	0.880
**P-stage**								
IA	1		1		1		1	
IB	1.25(0.55–2.83)	0.596	1.39(0.7–2.74)	0.345	1.16(0.64–2.11)	0.616	1.08(0.54–2.15)	0.836
IIA	2.16(1.05–4.45)	0.036	1.95(1.03–3.7)	0.041	1.37(0.74–2.57)	0.320	1.69(0.86–3.33)	0.130
IIB	1.6(0.58–4.4)	0.361	1.48(0.64–3.41)	0.354	2.93(1.69–5.09)	<0.001	4.17(2.29–7.59)	<0.001
IIIA	5.3(2.49–11.28)	<0.001	4.36(2.28–8.3)	<0.001	4.2(2.34–7.53)	<0.001	6.68(3.43–12.98)	<0.001
**Vascular invasion**								
No	1		1		1		1	
Yes	2.84(1.61–5)	<0.001	2.14(1.34–3.44)	0.002	1.57(1.03–2.41)	0.037	1.67(1–2.78)	0.048
	**(E) CD8 High**		**(F) CD3 High**		**(G) CD8 Low**		**(H) CD3 Low**	
	**HR** **(95% CI)**	***P***	**HR** **(95% CI)**	***P***	**HR** **(95% CI)**	***P***	**HR** **(95% CI)**	***P***
**PDGFRβ**								
Low	1		1		1		1	
High	0.89(0.55–1.44)	0.626	1.17(0.77–1.79)	0.464	1.85(1.28–2.66)	<0.001	1.45(0.97–2.17)	0.071
**Gender**								
Female	1		1					
Male	2.19(1.29–3.74)	0.004	1.8(1.15–2.8)	0.010				
**Histology**								
SCC	1		1					
ADC	2.84(1.73–4.67)	<0.001	2.22(1.42–3.46)	<0.001				
NOS	1.36(0.5–3.68)	0.546	1.93(0.95–3.93)	0.069				
**ECOG**								
Normal	1		1					
Slightly reduced	1.88 (1.12–3.15)	0.017	1.29(0.83–2.02)	0.254				
In bed <50%	2.51 (0.97–6.52)	0.059	1.76(0.77–4)	0.179				
**P-stage**								
IA	1		1		1		1	
IB	1.33 (0.59–3.04)	0.493	1.45 (0.73–2.89)	0.292	1.17 (0.64–2.14)	0.610	1.09 (0.55–2.2)	0.798
IIA	2.36 (1.15–4.85)	0.020	2.05 (1.08–3.91)	0.029	1.42 (0.77–2.64)	0.261	1.75 (0.9–3.43)	0.102
IIB	1.92(0.69–5.31)	0.212	1.67(0.72–3.87)	0.228	2.91(1.67–5.05)	<0.001	3.92(2.12–7.26)	<0.001
IIIA	5.63(2.64–12.02)	<0.001	4.52(2.36–8.66)	<0.001	4.19(2.35–7.45)	<0.001	6.3(3.29–12.06)	<0.001
**Vascular invasion**								
No								
Yes	2.36(1.31–4.26)	0.004	2.01(1.23–3.31)	0.006	1.47(0.96–2.27)	0.078	1.53(0.93–2.53)	0.097

*Abbreviations*: FAP-1, Fibroblast activating protein 1; NSCLC, non small-cell lung cancer; CD8, cluster of differentiation 8; ECOG, Eastern Cooperative Oncology Group.

## Discussion

A major finding from this *in situ* study is that the levels of CAFs did not correlate markedly with the infiltration of major leukocyte subsets into NSCLC tumor tissue (Fig 2B), indicating that CAFs may not play a dominant role in the regulation of leukocyte recruitment/infiltration in these tumors. Interestingly, survival analyses show that high levels of CAF^FAP^ in CD3/CD8 infiltrated tumors correlate with increased patient survival. This finding may suggest that CAF^FAP^ positively influence the effector function of cytotoxic tumor infiltrating lymphocytes.

The utilization of a TMA approach, rather than whole tissue slides, may partly explain the lack of correlations between CAFs and leukocyte subsets. However, the potential negative effect of using TMA in this context should be negated by the sheer number of patients included in the study and the inclusion of duplicate cores from each patient leading to increased representativeness. In addition, the presence of phenotypically different subsets of CAFs may differ between tumor regions due to heterogeneity. Neither TMA, nor whole slide studies are able to fully address this issue. Double, triple or quadruple IHC staining would ensure intra-patient sample homogeneity and would allow co-localization of CAF markers on the same cells. However, the issue of inter-patient sample heterogeneity, due to structurally and functionally different tumor areas being investigated in each patient, will remain a problem. Moreover, the spatial organization of the immune cell infiltrate, which discriminates between peritumoral and intratumoral areas is not addressed in TMA-based analyses. This is a relevant point that should be considered at the time of interpreting the results from this study. In this work, we lack information on peritumoral areas or the invasive front of tumors. However, we have considered the distinction between stromal and intratumoral areas.

Tumor fibroblasts, or CAFs, refers to a heterogeneous population of mesenchymal cells occurring during tumor development. Each subclass express different sets of cellular markers, and probably exert different regulatory functions in the context of cancer [1]. Despite the well-defined set of markers to identify mesenchymal cells, it is not possible to differentiate CAF subtypes based merely on the overlapping or non-overlapping expression of these markers [35]. Hence, we selected a panel of frequently used markers of fibroblast activation, and analyzed them separately. Initially, we checked for correlations between CAF-markers to study CAF diversity in NSCLC (Table 1, Fig 2A). Notably, vimentin, PDGFRα, PDGFRβ and FAP-1 showed modest correlation and did not correlate with αSMA and MT (collagen). Hierarchical clustering indicate the existence of different CAF subsets. Intriguingly the expression patterns of FAP-1 and αSMA, two of the most frequently used markers of CAF activation, showed distinct expression patterns when the staining was conducted in the same tumor specimen (Fig 3), clearly demonstrating the existence of phenotypically different CAF subsets in NSCLC tumors. Additionally, FAP-1 was not exclusively expressed by CAFs in tumors; since positive expression was also found in macrophage like cells, as previously observed for other tumor types [36]. However, our scoring was restricted to FAP-1 expression in fibroblast-like cells. In addition, PDGFRα expression by neoplastic epithelial cells was observed in about 15% of the cases.

In our cohort of unselected stage I-IIIA NSCLC patients, we have previously studied more than 100 prognostic markers related to *in situ* immunology [37–39], angiogenesis [30, 34], and epithelial-mesenchymal transition [10]. In this study, we have capitalized on these previous analyses and checked for correlations between CAF-markers and a panel of different leukocyte markers in the same patient cohort. Overall, the different CAF-markers do not show strong correlations with any of the selected myeloid or lymphoid immune markers, which included CD3, CD4, CD8 (T-lymphocytes), CD20 (B-lymphocytes), CD68 (TAMs), CD1a (dendritic cells DCs), CD56 (NK cells), FoxP3 (Treg) and CD45RO (T-memory cells). Moderate positive correlations were observed between CAF^FAP^ and CAF^PDGFRβ^ with FoxP3, and between CAF^PDGFRα^ and CD3 or CD20 (Fig 2B). The positive associations of CAF subsets with T regulatory cells highlight a potential CAF-mediated immuno-regulatory mechanism connected to the chemotaxis of immuno-suppressive cells.

The interplay between CAFs and immune cells has been recognized as a major contributor in cancer development as summarized in recent reviews [23, 40]. CAFs may influence trafficking and function of effector immune cells indirectly by modulating ECM deposition and stiffness, via the synthesis of ECM components such as collagens, fibronectin and laminin, or by the release of matrix remodeling agents including MMPs, cathepsins or proteases of the uPA system [41, 42]. In our study we have identified correlations between CAF^αSMA^ and collagen deposition in tumors (by MT), indicating that CAFs expressing αSMA (myofibroblasts) may be the main cells responsible for ECM formation in NSCLC tumors. However, no clear correlations were found between MT or CAF^αSMA^ patterns and the infiltration of immune cells.

According to numerous pre-clinical studies, the pleiotropic immune-modulatory functions of CAFs are orchestrated primarily through the production of a plethora of cytokines, chemokines and small molecules functioning in a paracrine fashion [43, 44]. Moreover, CAFs have been shown to regulate recruitment and polarization of innate immune cells such as macrophages and neutrophils via secretion of factors like CCL5, MCP-1/CCL2, IL-6, IL-8, CXCL10 and CXCL14 [18, 45]. In the presented study, results do not reveal clear associations between CAFs and intratumoral levels of either TAMs (CD68) or DCs (CD1a). Whether CAFs influence the functional polarization of TAMs or DCs *in vivo*, remains to be elucidated. In future studies, the phenotypic stage of these cells should be established by the use of differentiation markers such as CD206 (M2 macrophages), iNOS (M1 macrophages) or CD303 (plasmacytoid DC).

Furthermore, CAFs regulate recruitment and activation of adaptive immune cells via the release of peptide signals such as transforming growth factor β1 (TGFβ1), tumor necrosis factor-α (TNFα), stromal-derived factor 1 (SDF-1), or thymic stromal lymphopoietin (TSLP) [46–49]. In our NSCLC patient cohort, we have investigated associations between CAF subtypes and lymphoid cells including T-cells (CD3^+^), CD4^+^ T-helper cells, CD8^+^ cytotoxic lymphocytes, memory T-cells (CD45RO), T-regulatory cells (FoxP3^+^), B-lymphocytes (CD20^+^) and NK cells (CD56^+^). No relevant correlations were observed between CAFs and adaptive immune cells. However, with the intention of checking CAF influence on T-cell effector functions, survival rates in patients with high or low infiltration rates of CD3^+^ and CD8^+^ T-cells were compared. Intriguingly, in the subgroup of patients with tumors highly infiltrated by lymphocytes, the presence of CAF^FAP^ was a positive prognostic factor. This observation suggests that CAF^FAP^ may—directly or indirectly—exert positive immuno-adjuvant effects. On the contrary, increased CAF^PDGFRβ^ levels were associated with adverse prognosis in patients with tumors poorly infiltrated by T-lymphocytes (S3 Fig). This latter finding indicate that CAF^PDGFRβ^ exerts no influence on the tumor immune response. Combined, the results of CAF^FAP^ and CAF^PDGFRβ^ support the theory of functionally different and/or context-dependent CAF subclasses exerting divergent immune-modulatory effects in NSCLC, despite the assumption that both FAP-1 and PDGFRβ are considered specific markers for activated CAFs. FAP-1, also known as seprase, is a ubiquitously expressed membrane bound serine protease that has both dipeptidyl peptidase and endopeptidase activities, cleaving substrates at post-proline bonds. FAP-1 is essentially undetectable in non-disease tissues, but greatly enhanced at sites of tissue remodeling, including fibrotic tissue reactions, chronically inflamed tissues, epithelial cancers and embryonic tissues. The role of this protease as regulator of the immune system is uncertain. However, selective depletion of FAP-1-positive fibroblasts in murine models of pancreatic cancer has been shown to enhance anti-tumor immunity and delay tumor growth [50].

Generally, CAFs have been considered to promote an immunosuppressive microenvironment, but this may be context dependent rather than a specific feature of CAFs. Most of the accumulated evidence in this regard are limited to *in vitro* experiments, or animal experiments with admixed fibroblasts previously expanded *in vitro* which do not recapitulate the CAF heterogeneity observed *in vivo*. Only a handful of studies, with diverging outcomes, have investigated immunoregulatory effects of CAFs *in vivo* by selective depletion of CAF subsets. In genetically engineered murine models, selective depletion of CAF^FAP^ showed antitumor effects via intratumoral recruitment of CD8+ T cells [50]. On the contrary, specific depletion of CAF^αSMA^ led to invasive tumors associated with an immunosuppressive adaptive response [25]. Besides, clinical studies in pancreas, breast and lung cancer that correlate ECM or CAF-markers with disease outcomes have shown that patients with high desmoplasia or CAF infiltration can have improved prognosis and overall survival [51]. The presented explorative study propose a beneficial prognostic role played by CAF^FAP^ in immune infiltrated tumors, and underscores the need of caution in targeting CAF^FAP^ as a therapeutic strategy in lung cancer. However, additional functional studies should be considered to conclusively demonstrate causal effects of CAF subtypes on the anti-tumor immune responses.

## Supporting information

S1 Fig **Immunostaining of TMA cores showing different scores for CD3 (A) and CD8 (B).** Abbreviations: CD, Cluster of differentiation.
(TIFF)

S2 Fig **Spearman's rank correlations** between (A and B). Different CAF markers and (C and D) CAF markers and markers of leukocyte subsets in the adenocarcinoma (A and C) and squamous cell carcinoma (B and D) subgroups. *P < 0.05, **P < 0.01, ***P < 0.001. Abbreviations: CAF, cancer-associated fibroblast; Vim, vimentin; FAP-1, Fibroblast activation protein 1; PDGFR, platelet-derived growth factor receptor; αSMA, alpha-smooth muscle actin; MT, Masson's trichrome; CD, cluster of differentiation.
(TIFF)

S3 Fig **Disease-specific survival curves** for: PDGFRα in patients expressing low levels of **A)** CD8 and **B)** CD3, PDGFRβ in patients expressing low levels of **C)** CD8 and **D)** CD3, FAP-1 in patients expressing low levels of **E)** CD8 and **F)** CD3 and αSMA in patients expressing low levels of **G)** CD8 and **H)** CD3. Abbreviations: FAP-1, Fibroblast activating protein 1; PDGFR, platelet-derived growth factor receptor; αSMA, alpha-smooth muscle actin.
(TIFF)

S1 Table Antibodies and staining conditions with ventana discovery-ultra instrument. (DOCX)

S1 File Data used for survival analyses. (CSV)
