# Supplementary figures and images for: Tissue analyses reveal a potential immune-adjuvant function of FAP-1 positive fibroblasts in non-small cell lung cancer
Source: PLoS One. 2018 Feb 7;13(2):e0192157. doi: 10.1371/journal.pone.0192157 (PMC5802915; doi:10.1371/journal.pone.0192157)

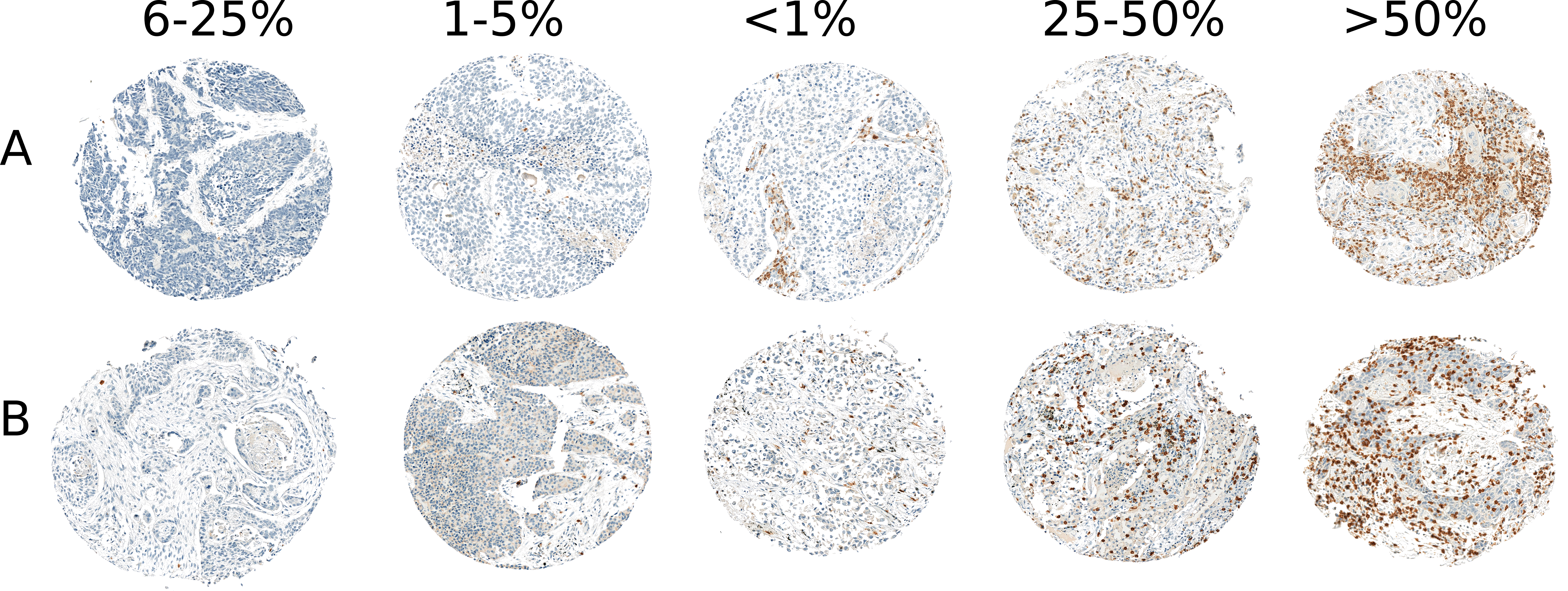

Supplement: S1 Fig — Immunostaining of TMA cores showing different scores for CD3 (A) and CD8 (B). Abbreviations: CD, Cluster of differentiation. (TIFF) [file pone.0192157.s001.tiff]

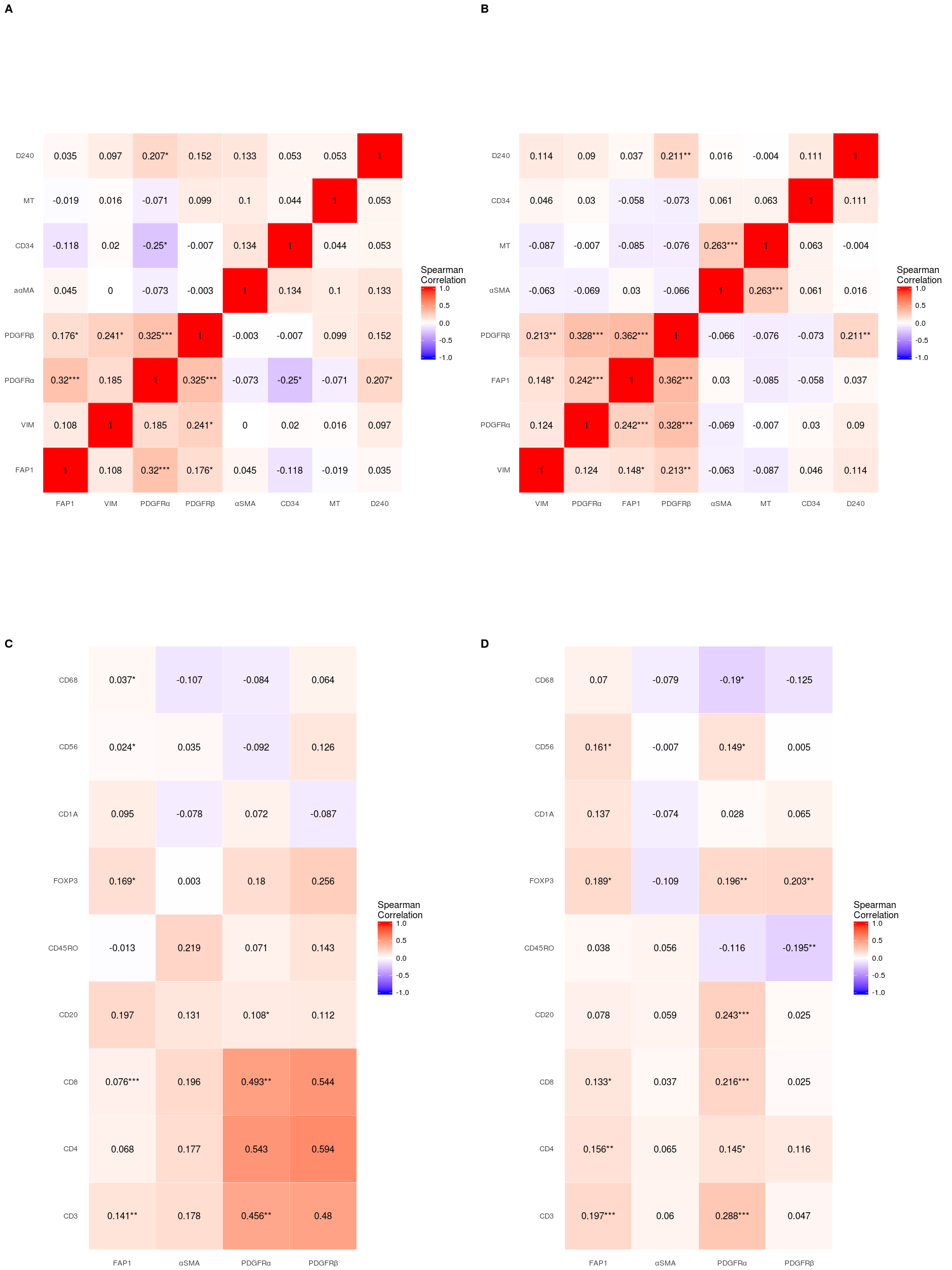

Supplement: S2 Fig — Spearman's rank correlations between (A and B). Different CAF markers and (C and D) CAF markers and markers of leukocyte subsets in the adenocarcinoma (A and C) and squamous cell carcinoma (B and D) subgroups. *P < 0.05, **P < 0.01, ***P < 0.001. Abbreviations: CAF, cancer-associated fibroblast; Vim, vimentin; FAP-1, Fibroblast activation protein 1; PDGFR, platelet-derived growth factor receptor; αSMA, alpha-smooth muscle actin; MT, Masson's trichrome; CD, cluster of differentiation. (TIFF) [file pone.0192157.s002.tiff]

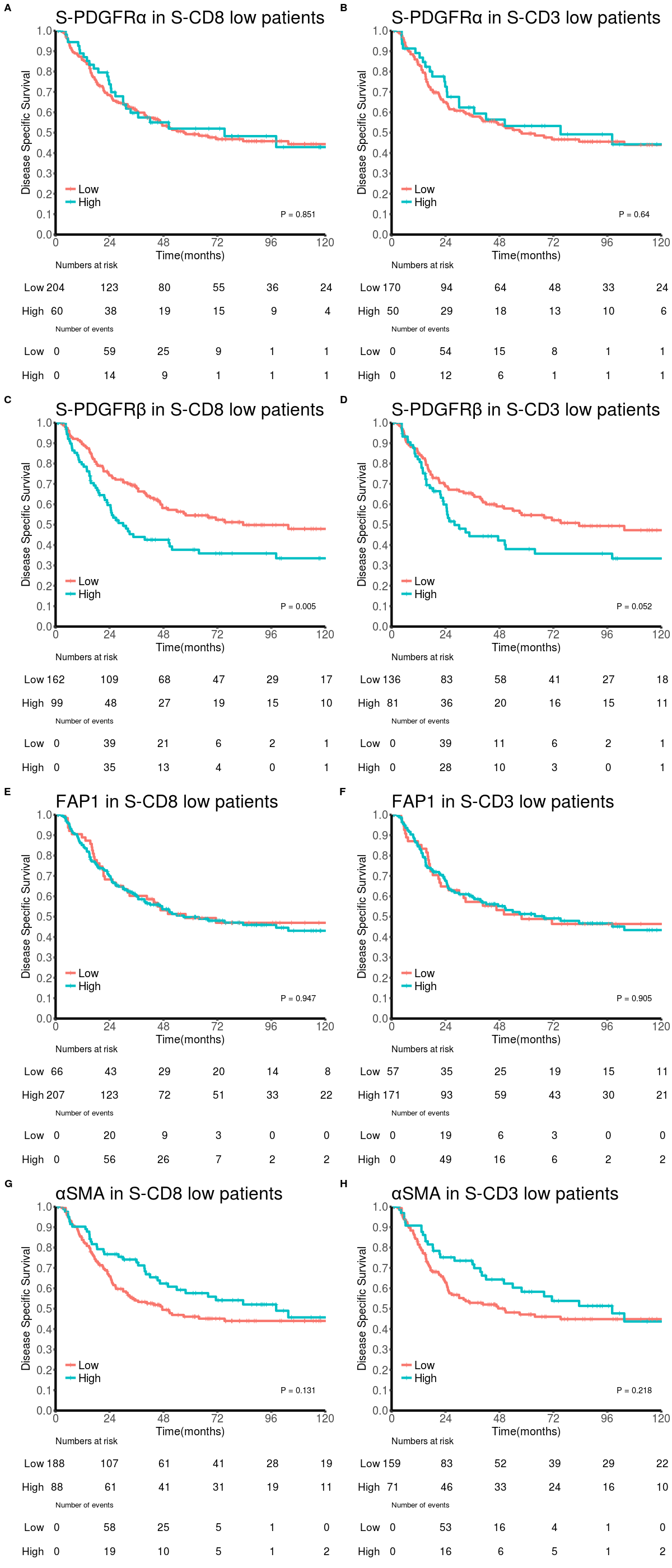

Supplement: S3 Fig — Disease-specific survival curves for: PDGFRα in patients expressing low levels of A) CD8 and B) CD3, PDGFRβ in patients expressing low levels of C) CD8 and D) CD3, FAP-1 in patients expressing low levels of E) CD8 and F) CD3 and αSMA in patients expressing low levels of G) CD8 and H) CD3. Abbreviations: FAP-1, Fibroblast activating protein 1; PDGFR, platelet-derived growth factor receptor; αSMA, alpha-smooth muscle actin. (TIFF) [file pone.0192157.s003.tiff]
