# Supplementary material for: Tissue analyses reveal a potential immune-adjuvant function of FAP-1 positive fibroblasts in non-small cell lung cancer
Source: PLoS One. 2018 Feb 7;13(2):e0192157. doi: 10.1371/journal.pone.0192157 (PMC5802915; doi:10.1371/journal.pone.0192157)
Supplement: S1 Table — (DOCX) [file pone.0192157.s004.docx]

S1 Table: Antibodies and staining conditions with Ventana Discovery-ultra instrument

| **Antibody** | **Clone** | **Source** | **Detection**  **system** | **dilution** |
| --- | --- | --- | --- | --- |
| PDGFRα | D13C6 | Cell signaling | DAB  Amp.HQ: 4 min | 1/100 |
| PDGFRβ | 28E1 | Cell signaling | DAB  Amp.HQ:12 min | 1/50 |
| FAP-1 | Polyclonal | Abcam | DAB | 1/25 |
| α-SMA | 1A4 | Ventana | DAB | Pre-diluted |
